# Antidepressant use during pregnancy and the risk of developing gestational hypertension: a retrospective cohort study

**DOI:** 10.1186/s12884-018-1825-y

**Published:** 2018-05-29

**Authors:** Neily Zakiyah, Loes F. ter Heijne, Jens H. Bos, Eelko Hak, Maarten J. Postma, Catharina C. M. Schuiling-Veninga

**Affiliations:** 1 Unit of PharmacoTherapy, -Epidemiology & -Economics (PTEE), Department of Pharmacy, University of Groningen, A. Deusinglaan 1, 9713 AV Groningen, The Netherlands; 2 Department of Epidemiology, University Medical Center Groningen, University of Groningen, 9713 GZ Groningen, The Netherlands; 3 Institute of Science in Healthy Aging & healthcaRE (SHARE), University Medical Center Groningen, 9713 GZ Groningen, The Netherlands

**Keywords:** Antidepressive agents, Pregnancy, Gestational hypertension, Preeclampsia

## Abstract

**Background:**

Prior studies reported that exposure to antidepressants during pregnancy may be associated with gestational hypertension. The aim of this study is to assess the association between the use of antidepressants during pregnancy and the risk of developing gestational hypertension.

**Methods:**

A retrospective cohort study using the prescription database IADB.nl was conducted among nulliparous women with singleton pregnancies between 1994 and 2015 in the Netherlands. Logistic regression analysis was used to estimate odds ratios (OR), adjusted OR (aOR) and their corresponding 95% confidence intervals (95% CI). Gestational hypertension as main outcome measure was defined as at least one dispensed record of an antihypertensive drug (methyldopa, nifedipine, labetalol, ketanserin, nicardipine) after 20 weeks of gestation until 14 days after delivery. Sub-analyses were conducted for class of antidepressant, duration and amount of use of antidepressant (≤30, ≥30 Defined Daily Doses or DDDs), and maternal age. Sensitivity analyses to assess uncertainties were conducted.

**Results:**

Twenty-eight thousand twenty women were included, of which 539 (1.92%) used antidepressants. The risk of gestational hypertension was doubled for women using antidepressant (aOR 2.00 95% CI 1.28–3.13). Significant associations were also found for the subgroup selective serotonin reuptake inhibitors (SSRIs) (aOR 2.07 95% CI 1.25–3.44), ≥30 DDDs (aOR 2.50 95% CI 1.55–3.99) and maternal age of 30–34 years (aOR 2.59 95% CI 1.35–4.98). Varying the theoretical gestational age showed comparable results.

**Conclusion:**

Prolonged use of antidepressants during the first 20 weeks of gestation appeared to be associated with an increased risk of developing gestational hypertension. When balancing the benefits and risks of using these drugs during pregnancy, this should be taken into account.

## Background

During pregnancy, approximately 14 to 23% of pregnant women suffer from depressive symptoms [1, 2]. In the Netherlands, it was estimated that almost 2% of pregnant women are exposed to antidepressants [3]. Next to potential benefits of avoiding risks of an untreated depression [4, 5], the use of antidepressants during pregnancy is also potentially associated with adverse maternal and neonatal outcomes. Current evidence suggests that antidepressants use during pregnancy may be associated with gestational hypertension and preeclampsia [6–12]. These disorders are a major cause of morbidity and mortality for both the mother and the offspring worldwide [13, 14], and imposes substantial burdens on the society, economy and healthcare system [15, 16]. Gestational hypertension and preeclampsia share similar symptom, clinically recognized as new-onset hypertension after twenty completed weeks of gestation, in women who used to be normotensive. Although, unlike gestational hypertension, preeclampsia is accompanied by proteinuria [17, 18]. Gestational hypertension is also a known risk factor for pre-eclampsia, as patients often later progress to pre-eclampsia [19].

The primary cause of gestational hypertension is still poorly understood, but it has been suggested that genetic, environmental, and other predisposing factors such as diabetes mellitus, anxiety, and depression may increase the risk of developing gestational hypertension [20, 21].

Whether antidepressants affect the risk of developing gestational hypertension, independently of the underlying disease itself, remains uncertain. Nevertheless, there is growing evidence that the use of antidepressants is associated with gestational hypertension or preeclampsia. A recent review suggested that compared to evidence of possible association between antidepressants use and pre-eclampsia, the evidence on potential relation of antidepressants and gestational hypertension is rather limited [19]. It was reported that the exposure to selective serotonin reuptake inhibitors (SSRIs), the most commonly used antidepressants among pregnant women, during the first and second trimester of the pregnancy may elevate the risk of gestational hypertension or preeclampsia. The adjusted relative risks were reported ranging between 1.05–3.16 for pre-eclampsia [6–8, 12] and 1.16–1.9 for gestational hypertension [8, 11, 22]. Other antidepressants, particularly serotonin-norepinephrine reuptake inhibitors (SNRIs) and tricyclic antidepressants (TCAs) were also reported to be associated with the increased risk of preeclampsia [6, 7, 12] and gestational hypertension [11].

In order to further assess the risk-benefit of antidepressants use during pregnancy and provide additional information regarding the safety of these medications for pregnant women, we conducted a retrospective cohort study to evaluate the extent to which the use of antidepressants during pregnancy may increase the risk of developing gestational hypertension.

## Methods

### Study design and setting

A retrospective cohort study was performed with a large mother-infant subset from the University of Groningen’s IADB.nl pharmacy prescription database, referred to as “pregnancy database” [23]. The IADB.nl database is a longitudinal database containing pharmacy-dispensing data from community pharmacies in the Netherlands from 1994 to 2015, including approximately 600,000 patients. Each prescription record contains information on the date of dispensing, the quantity and dose regimen, the number of days the drug is prescribed for, the number of defined daily dose (DDD), and the Anatomical Therapeutic Chemical code (ATC code). Each patient has a unique anonymous identifier; the date of birth and gender are known. As Dutch patients generally register at one community pharmacy, the database contains an almost complete overview of the individual’s medication prescription history, except for medication prescribed during hospitalization and over-the-counter drugs. The database is considered to be representative for the general Dutch population [23, 24].

In the pregnancy database, a validated method is used to link newborn children in the IADB.nl database to their parents [24]. Also, the database contains the child’s birth date and the theoretical conception date was estimated as the child’s birth date minus 273 days (i.e. gestational duration of 39 weeks).

### Eligible participants

In accordance with definition from Dutch Society of Obstetrics and Gynaecology guidelines, gestational hypertension was defined when the symptom appeared after 20 weeks of gestation.

Singleton pregnant women who were registered in the IADB.nl pregnancy database during the period of 1994 until 2015 were included in the study. Women had to be enrolled in the database, at least 6 months prior to the theoretical conception date. Since gestational hypertension in a previous pregnancy increases the risk of developing gestational hypertension [25], only the first known pregnancy in the database was included. Women using antithrombotic agents (ATC code: B01A) were excluded since low-molecular weight heparin is associated with a reduction in the risk of pre-eclampsia in women with thrombophilia and renal disease [20]. Low-dosage acetylsalicylic acid is also used for the prevention of pre-eclampsia, thus users are excluded as well [26].

Women using antidiabetic drugs (ATC code: A10) prior to conception and those with at least one dispensing record of the antihypertensive drugs i.e. thiazides (ATC code C03AA), β-blocking agents (ATC code C07A), ACE-inhibitors (ATC code C09A), angiotensin-II antagonists (ATC code: C09C) or calcium channel blockers (ATC code: C08CA) in the period of 6 months before conception until 20 completed weeks of gestation were also excluded, as both diabetes mellitus and chronic hypertension are risk factors for developing gestational hypertension [27–29]. As migraine disorders are also reported to be associated with the increased risk of developing gestational hypertension, pregnant women having prescriptions for medications to treat migraine disorders (ATC code: N02C) 6 months before conception until twenty completed weeks of gestation were also excluded [30, 31].

### Exposure

Exposure was defined as at least one dispensing record of an antidepressant (ATC code: N06A) between the theoretical conception date and 20 completed weeks of gestation, calculated as the date of birth minus 133 days (i.e. 39 weeks). We defined the non-exposed group as pregnant women that were without antidepressant prescriptions in the period of 6 months prior to the theoretical conception date (calculated as the theoretical conception date minus 181 days) until 20 completed weeks of gestation.

### Outcome

The outcome was determined by identifying dispensed antihypertensive drugs to treat gestational hypertension according to Dutch Society of Obstetrics and Gynaecology [32]. A woman was considered to have gestational hypertension when she had at least one prescription for methyldopa (ATC code: C02AB), nifedipine (ATC code: C08CA05), labetalol (ATC code: C07AG01), ketanserin (ATC code: C02KD01), or nicardipine (ATC code: C08CA04) between 20 completed weeks of gestation and 14 days after delivery. This particular time was chosen because gestational hypertension and pre-eclampsia is the most important reason for a first dispensing of hypertensive treatment within 14 days after delivery, as an initiation of treatment or continuation of treatment during hospital stay/delivery, by community pharmacy in The Netherlands.

### Covariates

The following covariates that potentially confound the association between maternal exposure to antidepressants and gestational hypertension were assessed: maternal age at delivery as well as other medications before conception and during pregnancy, i.e. the use of fertility treatment (ATC codes: H01CA, H01CC, G03GA, G03GB, L02AE02, L02AE04 [33, 34] and maternal antibiotic prescriptions (ATC code: J01) [35]. We also took into consideration potential underlying condition that might affect the risk for developing preeclampsia i.e. mood disorders [9, 30] and obesity [20], and used prescriptions of benzodiazepines (ATC codes: N03AE, N05BA, N05CD or N05CF) and lipid modifying agents (ATC code: C10) as proxies for aforementioned conditions.

### Statistical analysis

A multivariate logistic regression was performed to estimate the odds ratio (OR) and their corresponding 95% confidence intervals (95% CI) of the association between antidepressant exposure and gestational hypertension. In multivariate analysis, OR were adjusted for variables that were significantly associated with the outcome in univariate analyses, to assess if there was a significant difference in distribution (*p* < 0.05) in the frequency or means of the covariates between exposed and non-exposed. The distribution of covariates was measure with Pearson Chi-square test (for categorical variables) or the independent T-test (for continuous variables). To examine whether the association varied by type of antidepressant, we subsequently stratified different classes of antidepressants i.e. non-selective monoamine reuptake inhibitors/ tricyclic antidepressants or TCAs (ATC code: N06AA), selective serotonin reuptake inhibitors or SSRIs (ATC code: N06AB), non-selective monoamine oxidase inhibitors or MAOI (ATC code: N06AF), reversible inhibitors monoamine oxidase A or RIMA (ATC code: N06AG) and other antidepressants (ATC code: N06AX). We also stratified exposure by the total amount of antidepressants dispensed during pregnancy (≤30, ≥30 DDDs), the period of exposure (0–10 weeks,11–20 weeks, only the first 10 weeks, and both periods from 0 to 20 weeks) and maternal age (15–19, 20–24, 25–29, 30–34, and 40+ years old). All statistical analyses were conducted using IBM SPSS Statistics 23.

### Sensitivity analyses

Notably, the estimation of theoretical conception date could have been overestimated because there is an increased risk of late preterm birth among women with gestational hypertension or preeclampsia [36, 37]. Therefore, a sensitivity analysis was performed to assess whether variation in the estimation of theoretical conception date had a substantial impact on the results. In addition to estimation of gestational duration of 39 weeks (birth date minus 273 days), we also estimated results for gestational durations of 37 weeks and 35 weeks, corresponding to birth date minus 259 days and 245 days, respectively.

Additionally, there are several database-related uncertainties that might affect the results. Firstly, previous study has indicated that there has been a significant increase in the use of antidepressants among pregnant women specifically in the Netherlands, over the last decades [38]. Secondly, due to limited information on the actual use of the prescribed antidepressants and also indication for the prescriptions, confounding by indication might present in the estimation. In order to address these uncertainties, we conducted additional series of sensitivity analyses where we adjusted the multivariate model to calendar year, and also an analysis to minimize confounding by indication by comparing women exposed to antidepressants between the theoretical conception date and 20 completed weeks of gestation to women who were exposed to the drugs in the sixth month before theoretical conception date. We also assessed the exposure group with at least two dispensing records of antidepressants instead of one.

## Results

### Primary analysis

**Fig. 1:**
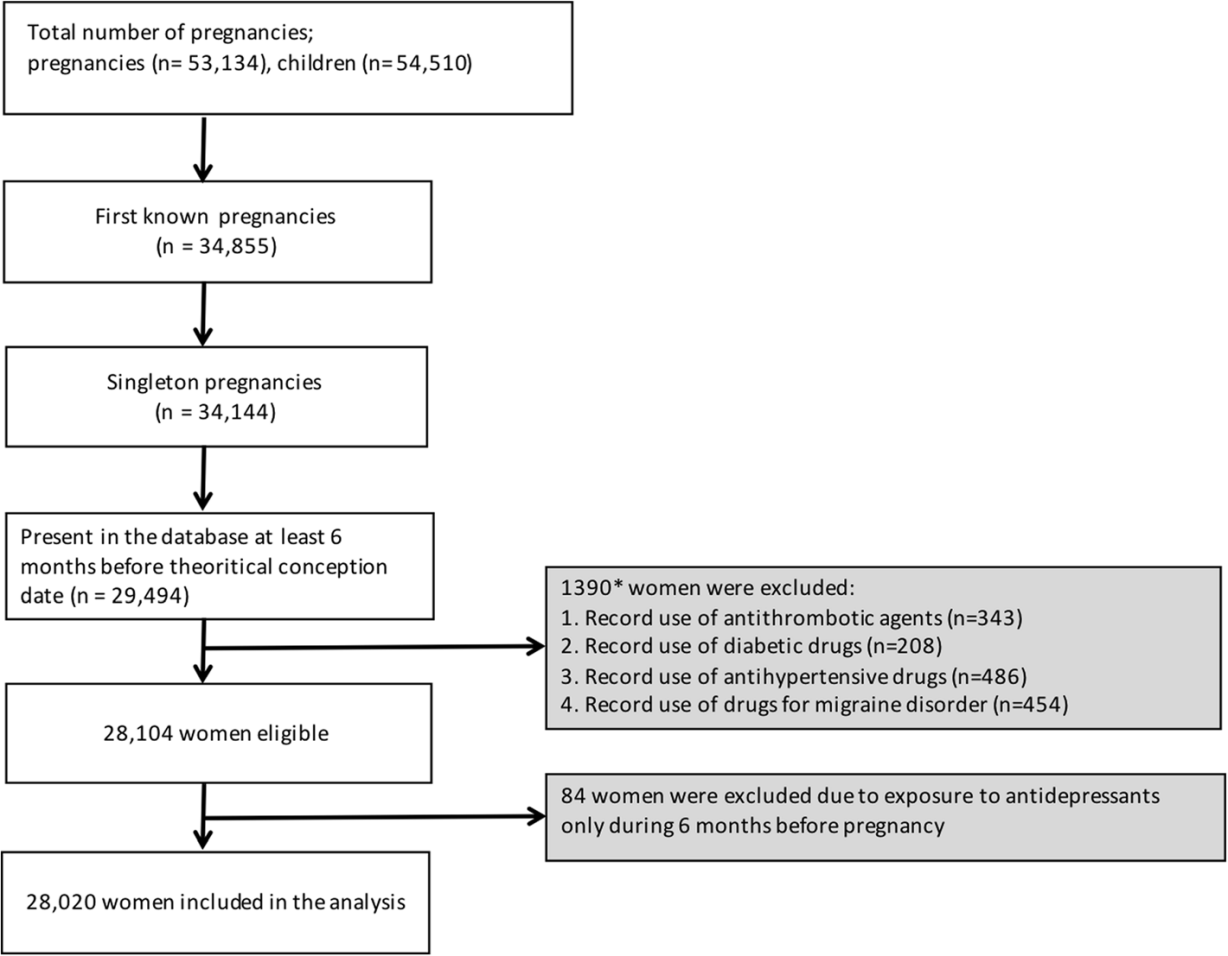
Flow diagram for participants’ selection in the analysis**.** *One woman could expose to multiple drugs listed in exclusion criteria

There were 28,020 pregnant women included in this cohort study (Fig. 1). Among these, 539 (1.9%) were exposed to antidepressants between the theoretical gestation date and twenty completed weeks of gestation. A detailed list of included antidepressants and number of pregnant women exposed is presented in Table 1. The majority of exposed women used SSRIs (73.10%) followed by TCAs (16.5%).

**Table 1:** Antidepressants included in the analysis and the number of pregnant women exposed

ATC-code	Medication^a^	Pregnant women (N)	Note
**N06AA**	**TCA**	**89**	
N06AA09	Amitriptyline	66	70 women used 1 type of antidepressant and 19 women used 2 different types of antidepressants from TCAs class
N06AA04	Clomipramine	31	
N06AA02	Imipramine	3	
N06AA10	Nortriptyline	3	
N06AA16	Dosulepin	2	
N06AA06	Trimipramine	1	
N06AA21	Maprotiline	1	
N06AA12	Doxepin	1	
**N06AB**	**SSRIs**	**394**	
N06AB05	Paroxetine	202	353 women used 1 type of antidepressant, 37 women used 2 different types of antidepressants and 4 women used 3 different types from SSRIs class
N06AB03	Fluoxetine	83	
N06AB04	Citalopram	75	
N06AB08	Fluvoxamine	45	
N06AB06	Sertraline	26	
N06AB10	Escitalopram	8	
**N06AF**	**MAOI**	**1**	
N06AF03	Phenelzine	1	–
**N06AG**	**RIMA**	**3**	
N06AG02	Moclobemide	3	–
**N06AX**	**Other**	**66**	
N06AX16	Venlafaxine	53	52 women used 1 type of antidepressant and 14 women used 2 different types of antidepressants from ‘other’ classes.
N06AX11	Mirtazapine	17	
N06AX21	Duloxetine	4	
N06AX05	Trazodone	3	
N06AX12	Bupropion	2	
N06AX06	Nefazodone	1	

*TCAs* tricyclic antidepressants, *SSRIs* selective serotonin reuptake inhibitors, *MAOI* non-selective monoamine oxidase inhibitors, *RIMA* reversible inhibitors monoamine oxidase

^a^One patient can be exposed to multiple antidepressants

The mean maternal age at delivery was 31.1 and 29.5 years for exposed and non-exposed pregnant women, respectively (see Table 2). The use of benzodiazepines and antibiotics were significantly higher amongst exposed compared to non-exposed women. In addition, there were no substantial differences in the use of lipid modifying agents and fertility treatment between both groups. Within the exposed group, 22 (4.1%) suffered from a medically treated gestational hypertension, whereas 571 (2.1%) of the non-exposed pregnant women were prescribed antihypertensive drugs in the period of twenty completed weeks of gestation until 14 days after delivery.

**Table 2:** Distribution of covariates in exposed and non-exposed pregnant women

Maternal characteristics	Exposed (*N* = 539)	Non-exposed (*N* = 27,481)	*P*-Value^a^
Mean maternal age at delivery (years)	31.10 ± 5.64	29.45 ± 4.77	<.001
Co-medication			
Benzodiazepines	151 (28.01%)	784 (2.85%)	<.001
Lipid Modifying Agents	3 (0.56%)	63 (0.23%)	.121
Antibiotics	321 (59.55%)	13,750 (50.03%)	<.001
Fertility Treatment	16 (2.97%)	1292 (4.70%)	.059
Cases of gestational hypertension	22 (4.08%)	571 (2.08%)	<.001

^a^*P*-value by Chi-squared test or T-test

After adjustment for maternal age, use of benzodiazepines, and use of antibiotics, the exposure to antidepressants during pregnancy was associated with significant increased odds for developing gestational hypertension (aOR 2.00, 95% CI 1.28–3.13; see Table 3). Analyses according to the class of antidepressants indicated that the risk of developing gestational hypertension was especially increased among women who used SSRIs (aOR 2.07, 95% CI 1.25–3.44). When examining exposure to different amounts of DDDs, the risk of developing gestational hypertension was only increased among women exposed to more than 30 DDDs of antidepressants, especially SSRIs (aOR 2.50, 95% CI 1.55–3.99 and aOR 2.27, 95% CI 1.44–3.60, for any type of antidepressants and specific SSRIs, respectively).

**Table 3:** Unadjusted and adjusted odds ratio for the development of gestational hypertension after exposure to antidepressant drugs during pregnancy in the primary analysis

Outcome	N	%	Unadjusted OR (95% CI)	*P*-Value	Adjusted OR (95% CI)^a^	*P*-value
Antidepressant use						
No exposure	27,481	98.08				
Exposure	539	1.92	1.99 (1.29–3.09)	.002	2.00 (1.28–3.13)	.002
Type of antidepressant*						
TCAs (N06AA)	89	16.51	1.61 (0.51–5.11)	.418	1.60 (0.50–5.09)	.429
SSRIs (N06AB)	394	73.10	2.11 (1.30–3.45)	.003	2.07 (1.25–3.44)	.005
MAOI (N06AF)	1	–	–	–	–	–
RIMA (N06AG)	3	–	–	–	–	–
Other (N06AX)	66	12.25	1.44 (0.35–5.90)	.611	1.43 (0.35–5.91)	.618
DDD						
≤ 30	150	27.64	0.64 (0.16–2.57)	.524	0.68 (0.17–2.75)	.584
≥ 30	389	72.36	2.55 (1.61–4.02)	<.001	2.50 (1.55–3.99)	<.001
DDD SSRIs						
≤ 30	91	16.70	0.52 (0.07–3.74)	.516	1.28 (0.56–2.92)	.561
≥ 30	303	56.40	2.61 (1.57–4.35)	<.001	2.27 (1.44–3.60)	<.001
Period						
0–10 weeks	506	93.88	2.14 (1.38–3.30)	.001	2.13 (1.36–3.34)	.001
11–20 weeks	290	53.80	2.38 (1.38–4.10)	.002	2.36 (1.35–4.12)	.003
0–10 weeks only	249	46,20	1.56 (0.77–3.17)	.220	1.59 (0.78–3.26)	.205
Both periods (0–20 weeks)	257	47.68	2.71 (1.57–4.67)	<.001	2.66 (1.52–4.65)	.001
Maternal age						
15–19	474	1.69	–	–	–	–
20–24	3636	12.98	1.11 (0.15–8.16)	.919	–	–
25–29	10,233	36.52	2.03 (0.89–4.65)	.093	–	–
30–34	9597	34.25	2.59 (1.35–4.98)	.004	–	–
34–39	3499	12.49	1.23 (0.38–3.95)	.731	–	–
40+	581	2.07	1.26 (0.29–5.56)	.758	–	–

*OR* odds ratio, *CI* confidence interval, *TCAs* tricyclic antidepressants, *SSRIs* selective serotonin reuptake inhibitors, *MAOI* non-selective monoamine oxidase inhibitors, *RIMA* reversible inhibitors monoamine oxidase A, *DDD* defined daily dose

^a^Adjusted for maternal age and medications use during pregnancy i.e. prescriptions of benzodiazepines and antibiotics

*One patient can be exposed to multiple antidepressants

The vast majority of women who used antidepressants during pregnancy (506 women out of 539), were exposed to the drugs during the first 10 weeks of the pregnancy, and more than half of them (257) continued the medication during the following 10 weeks of gestation. Prolonged exposure to antidepressants seemed to significantly increase the odds of developing gestational hypertension, with aOR of 2.13 (95% CI 1.36–3.34), 2.36 (95% CI 1.35–4.12) and 2.66 (95% CI 1.52–4.65) for exposure in the period of 0–10 weeks of gestation including both women with and without ongoing exposure,11–20 weeks of gestation, and both periods, respectively. However, discontinuation of the exposure after the first 10 weeks of gestation (or those without ongoing exposure) did not seem to be associated with increased risk of developing gestational hypertension.

When the analysis was stratified based on maternal age, it appeared that only the age group of 30–34 years old had an association of increased risk of gestational hypertension, while the other age groups did not show any significant associations (Table 3).

### Sensitivity analyses

The results from sensitivity analysis for theoretical gestational durations of 37 weeks and 35 weeks demonstrated similar results, with antidepressants being associated with increased risk of gestational hypertension, with aOR of 2.17 (95% CI 1.37–3.44) and 2.21 (95% CI 1.37–3.56), respectively. SSRIs remained as the one antidepressant that is being prescribed for the vast majority of women during their pregnancy and exposure to SSRIs remained associated with significantly elevated odds of developing gestational hypertension with aOR of 2.14 (95% CI 1.28–3.60) for 37 weeks and 2.09 (95% CI 1.20–3.64) for 35 weeks. As expected, the sub-analysis results also showed rather similar results. Details for sensitivity analysis for the duration of gestation are provided in Table 4.

**Table 4:** Unadjusted and adjusted odds ratios for the development of gestational hypertension after exposure to antidepressant in the sensitivity analysis for the duration of gestation

Outcome	37 weeks			35 weeks		
	Unadjusted OR (95% CI)	Adjusted OR (95% CI)^a^	*P*-Value	Unadjusted OR (95% CI)	Adjusted OR (95% CI)^a^	*P*-value
Antidepressant use						
No exposure	–	–	–	–	–	–
Exposure	2.13 (1.36–3.32)	2.17 (1.37–3,44)	.001	2.14 (1.34–3.42)	2.21 (1.37–3.58)	.001
Type of antidepressant						
TCAs	1.92 (0.60–6.11)	1,98 (0.62–6.34)	.252	2.20 (0.69–7.02)	2,31 (0.72–7.44)	.161
SSRIs	2.15 (1.29–3.57)	2.14 (1.28–3.60)	.004	2.08 (1.21–3.57)	2.09 (1.20–3.64)	.009
MAOI	–	–	–	–	–	–
RIMA	–	–	–	–	–	–
Other	1.62 (0.39–6.63)	1.64 (0.40–6.78)	.495	1.92 (0.47–7.92)	1.98 (0.48–8.23)	.348
DDD						
≤ 30	0.72 (0.18–2.90)	0,79 (0.19–3.23)	.744	1.35 (0.43–4.27)	1.45 (0.46–4.62)	.529
≥ 30	2.68 (1.68–4.29)	2.65 (1.63–4.29)	<.001	2.41 (1.45–4.01)	2.44 (1.45–4.11)	.001
DDD SSRIs						
≤ 30	0.54 (0.07–3.90)	0.59 (0.08–4.31)	.607	1.48 (0.36–6.06)	1.57 (0.38–6.49)	.532
≥ 30	2.68 (1.68–4.29)	2.65 (1.63–4.28)	.001	2,23 (1.24–3.99)	2.20 (1.21–3.98)	.010
Period						
0–10 weeks	2.21 (1.40–3.49)	2.25 (1.41–3.61)	.001	2.32 (1.43–3.74)	2.38 (1.46–3.90)	.001
11–20 weeks	2.48 (1.47–4.20)	2.48 (1.45–4.26)	.001	2.42 (1.43–4.10)	2.44 (1.43–4.20)	.001
0–10 weeks only	1.56 (0.69–3.54)	1.64 (0.71–3.74)	.242	1.50 (0.55–4.07)	1.59 (0.58–4.35)	.365
Both periods (0–20 weeks)	2.69 (1.56–4.64)	2,69 (1.53–4.68)	.001	2.74 (1.59–4.73)	2.75 (1.58–4.82)	<.001
Maternal age						
15–19	–	–	–	–	–	–
20–24	1.31 (0.18–9.68)	–	.790	1.77 (0.24–13.15)	–	.579
25–29	1.97 (0.80–4.86)	–	.143	1.75 (0.64–4.78)	–	.278
30–34	2,88 (1.49–5.53)	–	.002	2.89 (1.45–5.75)	–	.003
34–39	1.29 (0.40–4.17)	–	.666	1.37 (0.42–4.41)	–	.600
40+	1.30 (0.29–5.73)	–	.729	1.30 (0.29–5.73)		.729

*OR* odds ratio, *CI* confidence interval, *TCAs* tricyclic antidepressants, *SSRIs* selective serotonin reuptake inhibitors, *MAOI* non-selective monoamine oxidase inhibitors, *RIMA* reversible inhibitors monoamine oxidase A, *DDD* defined daily dose

^a^Adjusted for maternal age and medications use during pregnancy i.e. prescriptions of benzodiazepines and antibiotics

The series of additional sensitivity analyses with adjustment to calendar year and alternative assessment regarding exposure in the comparison group showed that the odds ratios in the primary analysis changed with these variations. The above analyses suggested that antidepressants were still associated with an increased risk of gestational hypertension with aOR of 1.70 (95% CI 1.08–2.66) for adjustment to calendar year. However, in the analysis with an alternative comparison regarding exposure, the association was still appeared but no longer statistically significant (aOR:1.45 (95% CI 0.63–3.33). Nevertheless, analysis with exposure to at least two dispensing records of antidepressants resulted in similar outcome as the primary analysis with an aOR of 2.19 (1.40–3.43). Table 5 depicts the results of these estimations.

**Table 5:** Association between the development of gestational hypertension after exposure of antidepressant in the series of additional sensitivity analyses

Outcome	Adjusted OR (95% CI)^a^	*P*-value
Primary analysis		
Antidepressant use		
No exposure		
Exposure	2.00 (1.28–3.13)	.002
Additional sensitivity analyses		
Adjusted to calendar year*	1.70 (1.08–2.66)	.021
Alternative comparison regarding exposure**	1.45 (0.63–3.33)	.380
Exposure to at least two dispensing records^Ψ^	2.19 (1.40–3.43)	.001
Exposure to at least two dispensing records^Ψ^ and adjusted to calendar year*	1.84 (1.17–2.89)	.008

OR, odds ratio

^a^Adjusted for maternal age and medications use during pregnancy i.e. prescriptions of benzodiazepines and antibiotics

*Categorized into 1995–1999, 2000–2004, 2005–2009, and 2010–2015

**Women exposed to antidepressants between the theoretical conception date and 20 completed weeks of gestation were compared to women who were exposed to these drugs in the six-months period before theoretical conception date, but not during pregnancy

^Ψ^The analysis was restricted to women having at least two dispensing records of an antidepressants from theoretical conception date until 20 completed weeks of gestation

## Discussion

In this retrospective cohort, we observed that the odds of developing gestational hypertension were doubled among pregnant women who were exposed to antidepressants during their pregnancy compared to those without the exposure. Notably, the risks were even greater among women who exposed to SSRIs and with DDDs more than 30. Prolonged used of antidepressants during both first and second trimesters seemed to further increase this risk. Varying the theoretical gestation age and exposure to at least two dispensing records of antidepressants showed comparable results. The results did seem sensitive to adjustment to calendar year and variation in comparison group regarding exposure*.* Overall, these findings were in line with the results from previous studies, suggesting that the exposure of antidepressants during pregnancy is associated with higher risk of developing gestational hypertension or pre-eclampsia [6–8, 11, 12, 22, 39]. However, prior studies also reported conflicting results concerning the specific type of antidepressants that might be associated with the elevated risk of gestational hypertension and/or pre-eclampsia. Notably, several studies reported elevated risks for developing gestational hypertension and/or among women who exposed to SSRIs during their pregnancy [8, 11, 12, 39]. For example, Toh et al. suggested more than 200% increased risk for developing gestational hypertension with preeclampsia for women who continue to use SSRIs after their first trimester [8], while the other studies reported lesser elevated risk for either gestational hypertension or pre-eclampsia [11, 22, 39]. Two studies from Palmsten et al. [6, 7] reported that SSRIs might not be associated with increased risk of developing pre-eclampsia while exposure to other antidepressants (particularly SNRIs and TCAs) during pregnancy was associated with 50% up to 220% increased risk of developing pre-eclampsia. This discrepancy may be due to differences in determining the primary exposure window. Palmsten et al. only took exposure during the second trimester until the end of the first half of the third trimester into account, i.e. 90 until 225 gestational days and excluded women exposed to antidepressants during the first trimester, which could lead to underestimation of the risk. Despite the differences, our findings suggested that other classes of antidepressants may also be associated with increased risk of gestational hypertension, although statistically significant relationship was not observed. These findings were based on limited sample size, however still highlighted the possible association as well.

In addition, it has been known that some antidepressants such as those in “other” class i.e. venlafaxine and duloxetine, have a side effect of sustained elevation of blood pressure that was found to be clinically significant at high dosages [40]. Regardless, as we already stratified the data based on class of antidepressant, comparison of the medication with and without hypertension as an adverse effect was assumed to have a comparable result.

Furthermore, our results suggested that the risks of developing gestational hypertension were greater among women with DDDs more than 30. Higher DDDs in the analysis could mean polytherapy with multiple antidepressants. Moreover, as our database contains only information about dispensed prescriptions, there is a possibility that women who receive antidepressants only one time, did not really use the medication. When a woman receives an antidepressant more often during pregnancy, we can be more certain about real exposure to this medication.

Our current results also indicated that the age group of 30–34 years old had an increased risk of gestational hypertension. It is known that gestational hypertension is associated with advanced maternal age [41, 42]. A previous study reported that there was an increased risk of gestational hypertension for mothers who were more than 30 years old and that the risk was higher in older groups [41]. Another study suggested that the risk of pre-eclampsia was increased by approximately 4% for every year for mothers who were more than 32 years of age [42]. Due to our limited size in older age groups, we could not reproduce these results.

We also found that prolonged exposure of antidepressants during first and second trimesters may be associated with increased risk of developing gestational hypertension, but not for those who discontinued the treatment after the first trimester. This finding was in line with the study by Toh et al. [8] which documented that continuation of antidepressants after the first trimester might be associated with a higher risk for either gestational hypertension or preeclampsia when compared with discontinuation of the exposure during the first trimester of pregnancy. A recent study also reported similar patterns, suggesting that continuers in the second half of pregnancy were significantly associated with increased risk for gestational hypertension [43]. Based on inconsistencies both in methodology and results in previous studies, it was concluded that, although the relation of antidepressant use during pregnancy and the increased risk of developing gestational hypertension or pre-eclampsia is implied in these studies, current evidence might be inadequate to evidence a definite association, and suggests that definitive conclusion ideally requires further randomized controlled trials [19]. However, as it is considered unethical to perform randomized controlled trials in pregnant women to assess the relationship between maternal exposures and its related effects, only further series of adequately designed and performed observational studies can be used to inform such associations.

The stratification based on the period of exposure from conception date might give another perspective, highlighting that prolonged exposure during pregnancy may elevate the risk of gestational hypertension. Although the mechanism behind the association between antidepressants and gestational hypertension is still uncertain, it has been hypothesized that hypertension is a result of an imbalance in vasoconstrictors over vasodilators, that could be triggered by anxiety, stress, depression and –among other things- pharmacological interventions [8, 44].

Our study provides further evidence to the existing literature regarding the risk of antidepressants use during pregnancy and the association with adverse maternal health outcomes. The strength of this study was that we obtained the data from widely researched pharmacy-dispensing database with proven accuracy in the prescription rates, therefore recall bias of the drug use was likely eliminated. The database also contained a large sampled population with the possibility to observe the prescription for a long period of time (1994–2015). As the relevant treatment guidelines as well as available treatments remained unchanged in the Netherlands, we considered the aforementioned time period as sufficient to still represent the present conditions. Yet, we did a sensitivity analysis with adjustment to calendar year with the assumption that there was increasing trend in antidepressants exposure in pregnancy in the last decades [38].

Additionally, the database provided an almost complete overview of the individuals’ medication prescription, allowing us to take certain co-medications into account as possible confounders [23]. We also conducted a sensitivity analysis for the estimation of theoretical conception date. This analysis allowed us to limit misclassification in the early exposure of antidepressants and eliminate the overestimation of the risk. In the study design, we also considered the prescription of benzodiazepines as a proxy for mood disorder. Benzodiazepines were assessed because it remains unclear if psychotropic medications affect the risk of developing gestational hypertension, independently of mood disorders [9, 30]. This design allowed us to adjust and exclude the possibility that the increased risk of developing gestational hypertension was due to underlying mood disorder itself.

Our study has potential limitations. Although IADB.nl reflects a large follow-up prescription database, we did not have any information on the indication of the prescribed drugs nor the actual use of the drugs. The information of disease severity was also basically absent. In the analysis, we decided to make a clear distinction between exposure and no exposure, with women receiving a prescription for an antidepressant after theoretical conception date being considered as exposed and women without any prescription for an antidepressant as non-exposed. We also decided to exclude women receiving an antidepressant during the 6 months before conception. This also excludes women who choose to discontinue the medication as soon as they were aware of the pregnancy. Nevertheless, as an additional way to rule out potential confounding by indication, we did sensitivity analysis comparing women exposed to antidepressants during the relevant time window in pregnancy to those who were exposed to the drugs in the 6 months before theoretical conception date. The outcome showed that the result was sensitive to changing this definition of exposure and non-exposure. Confounding by indication could be an explanation for these results and future research needs to focus on disentangling the effect of antidepressants from the effect of the underlying depression. Beside this, a great deal of uncertainty remains around medication exposure during pregnancy, especially concerning the optimal way to classify exposure and non-exposure groups. Therefore, there is a need for more attention regarding this matter to minimize potential classification biases [45].

Additionally, we also could not distinguish between gestational hypertension and preeclampsia based on the available data. As women with the diagnosis of preeclampsia would be more likely to be admitted to the hospital for delivery or expectant management [20], the women who included in this study were more likely to have gestational hypertension without proteinuria.

In the analysis, we included all eligible pregnant women with at least one dispensing of antihypertensive drugs, as the prescription of these particular drugs during the second part of pregnancy could be considered to be due to hypertensive problems with great certainty. In more severe cases, when the antihypertensive medication is insufficient, the pregnant women will likely be admitted to the hospital. However, the database did not cover information of medication dispensed during hospitalization, which may have led to underestimation of the actual number of cases. We tried to overcome this by observing the outcome of gestational hypertension until 14 days after the delivery. With this timeframe, women who were hospitalized and received antihypertensive treatment afterwards, would still be partially visible in our analysis. The database lacks information on demographic or personal characteristics of the patients, which might potentially confound the association between the use of antidepressants and gestational hypertension. Moreover, we attempted to include relevant covariates that likely be potential confounders for the association. However due to the database-related limitation, it is impossible to assess all possible confounders in the analysis. For instance, uncontrolled asthma seems to be associated with increased risks of gestational hypertension and pre-eclampsia [46, 47]. However, due to lack of information on clinical status and actual drug use, it would be very difficult to distinguish controlled from uncontrolled asthma. This is particularly important, because a previous study has reported that there is no significant increased risk of gestational hypertension for users of inhaled corticosteroids or those with controlled asthma [46]. Regarding these limitations on the database, further research based on databases containing more pregnancy-specific and clinical information is advised to confirm our current findings.

Furthermore, SSRIs were the most prescribed antidepressants in our analysis. Consequently, other types of antidepressants may be confronted with a relative lack of statistical power to show associations. In the analysis, TCAs and other antidepressants showed increased risks although the relations were not statistically significant.

Lastly, while pharmacy database has been widely used for research, the data were actually limited to drugs dispensing, where the information whether the medications were actually taken was simply beyond the observation. Another constraint was that the database did not contain information about specific characteristics of patients such as body mass index (BMI), smoking status, alcohol use, socioeconomic status etc.

## Conclusion

This study suggests that exposure to antidepressants during pregnancy is associated with an increased risk of gestational hypertension. Since previous studies showed conflicting results, further observational studies based on more comprehensive databases containing complete demographic and clinical information are needed to further confirm our findings. They should also further focus on whether there is indeed a difference in the risk between types of antidepressants, time of exposure and whether the risk is due to the underlying depression. In deciding on antidepressants use in pregnancy, potential benefits as well as risks of antidepressants should both be considered during pregnancy, explicitly been taken into account and adequately discussed during pregnancy.

## Abbreviations

aOR: Adjusted odds ratios
ATC code: Anatomical Therapeutic Chemical code
CI: Confidence intervals
DDDs: Defined daily doses
MAOI: Non-selective monoamine oxidase inhibitors
OR: Odds ratios
RIMA: Reversible inhibitors monoamine oxidase
SNRIs: Serotonin-norepinephrine reuptake inhibitors
SSRIs: Selective serotonin reuptake inhibitors
TCAs: Tricyclic antidepressants
